# Association of sun exposure and seasonality with vitamin D levels in Brazilian children and adolescents

**DOI:** 10.1590/1984-0462/2023/41/2021361

**Published:** 2023-03-03

**Authors:** Polyana Romano Oliosa, Eduardo Magno Romano Oliosa, Rafael de Oliveira Alvim, Carmem Luiza Sartório, Divanei dos Anjos Zaniqueli, José Geraldo Mill

**Affiliations:** a Universidade Federal do Espírito Santo, Vitória, ES, Brazil.; b Instituto Federal do Espírito Santo, Vitória, ES, Brazil.; c Universidade Federal do Amazonas, Manaus, AM, Brazil.

**Keywords:** Vitamin D, Children, Adolescent, Season, Epidemiology, Vitamina D, Criança, Adolescente, Sazonalidade, Epidemiologia

## Abstract

**Objective:**

This study aimed to verify vitamin D concentration in children and adolescents during the seasons of the year and to compare vitamin D concentration between children engaged in outdoor activities and those engaged in indoor activities.

**Methods:**

This is a cross-sectional study with a sample of 708 children and adolescents (aged 6–18 years), excluding 109 (16 were over 19 years old; 39 had a disease that required continuous treatment; 20 were on continuous medication; and 34 had no vitamin D data), ending with 599. The plasma concentration of 25-hydroxyvitamin D2 was measured with commercial kits following manufacturer instructions.

**Results:**

Participants who engaged in outdoor activities, as well as those who had data collected during summer and spring, had higher levels of vitamin D. According to the Poisson regression, the proportion of participants with inadequate levels of vitamin D was greater in the participants whose vitamin D was measured during spring (PR 1.15, 95%CI 1.03–1.29) and winter (PR 1.18, 95%CI 1.05–1.32). Also, a greater proportion of inadequate vitamin D was observed for those engaged in indoor activities (PR 1.08, 95%CI 1.01–1.15).

**Conclusions:**

Participants who measured the vitamin during the summer and autumn had a lower prevalence of hypovitaminosis D. Even in regions with high solar incidence throughout the year, vitamin D levels can vary significantly during the period’s seasons.

## Resumo

**Objetivo:**

Verificar a concentração de vitamina D em crianças e adolescentes durante as estações do ano e comparar essa concentração entre crianças praticantes de atividades ao ar livre e aquelas praticantes de atividades em ambiente fechado.

**Métodos:**

Trata-se de estudo transversal com amostra de 708 crianças e adolescentes (seis a 18 anos), excluindo-se 109, pois 16 eram maiores de 19 anos; 39 tinham doença que exigia tratamento contínuo; 20 estavam em uso de medicação contínua; e 34 não tinham dados de vitamina D. Terminou-se, assim, com 599 pacientes. A concentração plasmática de 25-hidroxivitamina D2 foi medida com *kits* comerciais, seguindo as instruções do fabricante.

**Resultados:**

Os participantes que realizaram atividades ao ar livre, assim como aqueles que tiveram dados coletados durante o verão e a primavera, apresentaram níveis mais elevados de vitamina D. De acordo com a regressão de Poisson, a proporção de participantes com níveis inadequados de vitamina D foi maior naqueles cuja medição foi realizada durante a primavera (razão de prevalência — RP 1,15, intervalo de confiança — IC95% 1,03–1,29) e o inverno (RP 1,18, IC95% 1,05–1,32). Além disso, maior proporção de vitamina D inadequada foi observada para aqueles envolvidos em atividades internas (RP 1,08, IC95% 1,01–1,15).

**Conclusões:**

Participantes que mediram a vitamina durante o verão e o outono tiveram menor prevalência para hipovitaminose D. Mesmo em regiões com alta incidência solar ao longo do ano os níveis de vitamina D podem variar significativamente durante as estações.

## INTRODUCTION

Brazilian studies demonstrate a high prevalence of low vitamin D concentration in children and adolescents.^1–4^ Due to its activity in human metabolism, vitamin D is also categorized as a hormone.^5,6^ It acts in several metabolic pathways, such as the development of muscle mass and bones^7^ and influencing the concentration of lipid fractions,^8^ blood pressure,^9^ and other cardiovascular factors.^10,11,12^ Exposure of the skin to solar ultraviolet (UV) radiation is the elementary mechanism of vitamin D synthesis in humans.^13,14^

In our species, only 10–20% of the vitamin D necessary for proper function of the organism comes from diet. The central dietary sources are vitamin D3 (cholecalciferol, of animal origin, present in oily fish from cold and deep water, such as tuna and salmon) and vitamin D2 (ergosterol, of plant origin, present in edible fungi). The remaining 80–90% depends on endogenous synthesis,^15^ which mainly requires ultraviolet B (UVB) radiation at wavelengths between 290 and 315 nm.

Due to the position of the axis where the Earth translates around the sun, the more a location is displaced from the Equator, the greater the thickness of the atmospheric layer that sunlight must pass through, which causes attenuation in various wavelengths, including UVB radiation. This incidence angle of sunlight on Earth (solar zenith) also changes over the seasons, being greater in winter, when the amount of UVB rays reaching the Earth’s surface is lower.^16^

Therefore, vitamin D synthesis in the human body depends on a number of causes, including geographical factors (e.g., solar elevation, ozone, cloudiness, and albedo), individual human factors, such as skin darkness, outdoor activities, and age, as well as genetic factors.^17^

Sola et al.^18^ showed that in the solar hours between 10:00 and 14:00, vitamin D production reaches higher values in relation to the rest of the day. At these times, greater irradiance is also available, as the zenith angle is smaller, which means that the irradiance suffers less interference from the atmosphere.

No Brazilian study has focused on the influence of time spent with outdoor activities or the seasonality on the level of vitamin D. Thus, the aim of this study was to verify vitamin D concentration in children and adolescents during the seasons of the year and to compare vitamin D concentration between children engaged in outdoor activities and those engaged in indoor activities.

## METHOD

This is a cross-sectional, descriptive, and quantitative study including 599 children and adolescents, aged 6–18 years old, enrolled in public schools in the municipality of Serra/ES (latitude: 20°7’46”S/ longitude: 40°18’29” West). All participants also attended a social project named “Estação Conhecimento” (Knowledge Station) designed to offer complementary academic, cultural, and sports activities. The present work is part of more comprehensive research entitled “Determinants of elevated blood pressure in children and adolescents of different ancestry.” This is a sample of children and adolescents enrolled in the institution called Estação Conhecimento. The plan was that all participants enrolled in the institution from July 2018 to December 2020 would be invited to participate (n=1000). However, with the COVID-19 pandemic, the collection took place from July 2018 to November 2019, and 708 participants of the institution were invited to the study. Among them, 199 were excluded: 16 participants were over 19 years old, 39 had a disease that required continuous treatment, 20 were on continuous medication, and 34 had no vitamin D data. Therefore, the final sample had 599 participants.

A dedicated staff including nutritionists, nurses, and psychologists developed a semi-quantitative form with questions on demographic, socioeconomic, health, lifestyle, and physical activity aspects. The form was applied by a trained interviewer on the same day as blood was withdrawn (BD Vacutainer/EDTA). Blood samples were centrifuged in the same place as its collection, and plasma aliquots were sent to a central laboratory (Laboratório Tommasi) to measure plasma concentration of vitamin D. The plasma concentration of 25-hydroxyvitamin D2 was measured with commercial kits following the manufacturer’s instructions.

The 25-hydroxyvitamin D classification was performed as follows: vitamin D deficiency defined as 25(OH)D below 20 ng/mL, insufficiency in 25(OH)D of 21–29 ng/mL, and sufficiency in 25(OH)D above 30 ng/mL.^19^ For the analyses, dichotomous vitamin D was used, with ≥30 ng/mL being considered adequate and below <30 ng/mL inadequate.

Body mass index (BMI) was calculated as the ratio between weight and height squared (kg/m^2^). BMI was classified according to the standards of the World Health Organization (WHO).^20^

As this is a cross-sectional study, it is noteworthy that the classifications of the seasons of the year were determined according to the months of data collection. Thus, the classification of seasons was stratified as follows: summer, taking into account the months of December, January, and February; autumn, the months of March, April, and May; winter, the months of June, July, and August; and spring, the months of September, October, and November.^21^

Children and adolescents answered the following question on the questionnaire: “What activity do you perform at the Estação Conhecimento?” The possible answers were related to sports and cultural modalities, being carried out indoors (judo and music) and outdoors (swimming, athletics, sports initiation, and soccer). These children and teenagers spent the entire period in activity, inside the classroom or outside, with an average of 3 h without contact with the sun (when activities were conducted indoors) or in contact with the sun (when activities were outdoors), and the frequency of going to the institution was 3 days a week. Race/color was classified according to the Brazilian Institute of Geography and Statistics.^22^ All the children were of low socioeconomic class, and there was no measurement of food consumption related to vitamin D and no child was taking vitamin D replacement (the questionnaire had questions about medications and supplements used).

The database construction and statistical analyses were done using the SPSS for Windows, version 20.0, statistical software package. For descriptive analysis, the chi-square test was used and comparison between groups used ANCOVA adjusted for confounding variables. The association among vitamin D deficiency, seasons, and sun exposure was determined by Poisson regression with robust variance, expressed as prevalence ratios (PR) and confidence intervals of 95%.

To make the maps, the ArcGIS program was used with data from the NISR (National Institute for Space Research, Brazil) database, from the year 2018, version 10.7, which expressed the available solar radiation (Wh/m^2^/day) in the State of Espírito Santo, making it possible to visualize the changes in solar incidence in the four seasons of the year in ES and also in the Serra Municipality.

The present study is part of a more comprehensive research entitled “Determinants of high blood pressure in children and adolescents from different ancestry” and was approved by the institutional Human Research Ethics Committee (CAAE: 30385014.8.0000.5060, no. 725.488). Participants were invited and informed about the objectives of the study. Data collection was obtained after the written informed consent was signed by parents or guardians of all participants. Adolescents (12–19 years) also signed the assent form.

## RESULTS

The sample consisted of 599 children and adolescents aged 6–19 years, 42.1% female. Among the participants, 62 (10.4%) were classified with deficient vitamin D concentration, 257 (42.9%) insufficient, and 280 (46.7%) with sufficient. Thus, inadequate vitamin D concentration (<30 ng/mL) was observed in 53.3% (n=319) of the participants. Of the total, 413 (74.28%) participants engaged in outdoor activities. Even so, it was observed that 53.3% (n=319) of the participants presented inadequate vitamin D levels.

Table 1 shows the distribution of variables by vitamin D concentration. Noteworthy, winter was the season that vitamin D reached the highest proportion of inadequacy, whereas summer was the season with the lowest proportion.

**Table 1.** Description of the sample stratified by vitamin D status.

	Total	Adequate	Inadequate	p-value
	n (%)	n (%)	n (%)	
Seasons				
Spring	154 (25.7)	63 (22.5)	91 (28.5)	**<0.001**
Summer	43 (7.2)	26 (9.3)	17 (5.3)	
Autumn	169 (28.2)	101 (36.1)	68 (21.3)	
Winter	233 (38.9)	90 (32.1)	143 (44.8)	
Sun exposure				
Indoor	143 (25.7)	55(20.3)	88 (30.9)	**0.004**
Outdoor	413 (74.3)	216 (79.7)	197 (69.1)	
Age range				
6–8	113 (18.9)	59 (21.1)	54 (16.9)	0.131
9–11	203 (33.9)	97 (34.6)	106 (33.2)	
12–14	211 (35.2)	99 (35.4)	112 (35.1)	
15–18	72 (12)	25 (8.9)	47 (14.7)	
BMI status				
Low weight	20 (3.3)	6 (2.1)	14 (4.4)	0.262
Eutrophy	436 (72.8)	213 (76.1)	223 (69.9)	
Overweight	110 (18.4)	213 (76.1)	63 (19.7)	
Obesity	33 (5.5)	14 (5.0)	19 (6.0)	
Race/color				
White	164 (27.4)	84 (30.0)	80 (25.1)	0.112
Black	178 (29.7)	72 (25.7)	106 (33.2)	
Brown	257 (42.9)	124 (44.3)	133 (41.7)	

Chi-square test. p-value significant when <0.05 (indicated in bold). The seasons of the year were classified according to the month of data collection. Activities: indoor (music and judo) and outdoor (swimming, soccer, athletics, sports initiation). Vitamin D breakpoints: adequate ≥30 ng/mL and inadequate below <30 ng/mL.

It was observed that the highest percentage of participants participated in the research during the winter season, 39.56% (n=220).

According to the Poisson regression, the proportion of participants with inadequate levels of vitamin D was 15% and 18% greater in the participants whose vitamin D was measured during spring and winter, respectively. Similarly, the proportion of children and adolescents with inadequate levels of vitamin D was 8% greater for those engaged in indoor activities (Table 2).

**Table 2.** Prevalence ratio of vitamin D inadequacy, according to seasons and sun exposure status.

	Crude		Adjusted	
	PR (95%CI)	p-value	PR (95%CI)	p-value
Seasons				
Summer	1	----	1	----
Autumn	1.01 (0.89–1.13)	0.933	1.01 (0.90–1.14)	0.989
Spring	1.14 (1.02–1.28)	**0.020**	1.15 (1.03–1.29)	**0.010**
Winter	1.16 (1.03–1.29)	**0.010**	1.18 (1.05–1.32)	**0.004**
Sun exposure				
Outdoor	1	----	1	----
Indoor	1.09 (1.03–1.16)	**0.003**	1.08 (1.01–1.15)	**0.020**

PR: prevalence ratio; 95%CI: 95% confidence interval. Vitamin D inadequacy: <30 ng/mL. Adjusted model: age range, race/color, and sex. p-values in boldface are significant.

Table 3 shows the mean concentration of vitamin D stratified by indoor and outdoor activities. Of the total sample participants, 413 (74.28%) practiced outdoor activities, and 143 (25.71%) practiced indoor activities. Even with the majority of the sample practicing outdoor activities, it was observed that 53.3% (n=319) of the participants had an inadequate concentration of vitamin D (below 30 ng/mL).

**Table 3.** The mean concentration of vitamin D stratified by indoor and outdoor activities.

Activities	Vitamin D (ng/mL)		p-value
	n	Mean (SD)	
Indoor	143	29.4 (9.68)	**0.034**
Outdoor	413	31.4 (9.35)	
Total	556	30.9 (9.51)	

*ANCOVA test (variable adjusted for age, sex, race/color, and seasons): p-value significant when ≤0.05 (indicated in bold). Activities: indoor (judo and music) and outdoor (swimming, athletics, sports initiation, and soccer).

**Figure 1.**
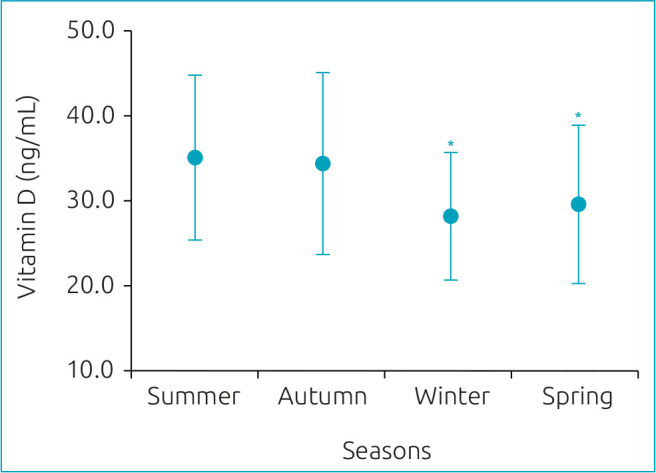
Vitamin D concentration according to the seasons. Marginal mean estimations adjusted for age, sex, race/color, and sun exposure status. ANCOVA test (variable adjusted for age, sex, race/color, and indoor and outdoor activities). *Significant lower than summer (p<0.05). Seasons stratification by months of the year: summer – December, January, and February; autumn – March, April, and May; winter – June, July, and August; spring – September, October, and November.

Figure 1 shows the mean values of vitamin D concentration according to the season. Interestingly, those children and adolescents who had vitamin D measured during winter (28.2±7.5 ng/dL and spring (29.6±9.3 ng/dL) presented lower vitamin D levels than those whose vitamin D was measured during summer (35.1±9.7 ng/dL).

**Figure 2.**
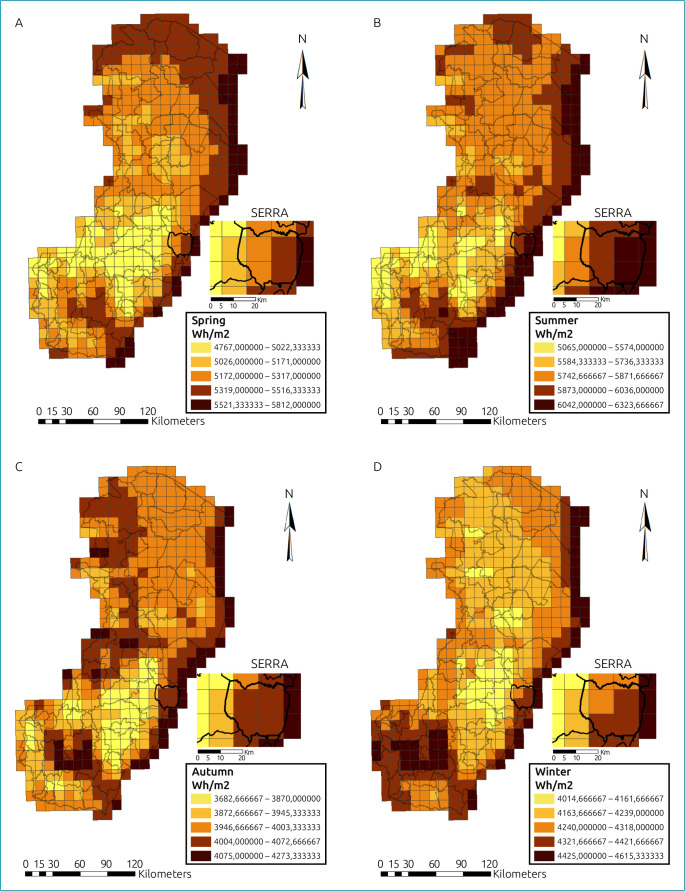
Changes of solar incidence in the seasons of the year in Espírito Santo. A: Spring; B: Summer; C: Autumn; D: Winter.

Figure 2 illustrates the maps showing the frequency of solar incidence throughout 2018, in the analysis carried out for the State of Espírito Santo, and this was stratified according to the four seasons of the year from the global horizontal radiation available in the geographical region analyzed in Watt-hour energy per square meter per day (Wh/m^2^/day).

It is noteworthy that the analysis was carried out based on 2018, and data collection took place from 2017 to 2019. However, as the Earth’s inclination does not vary much per year, the year described can be used as a basis.

The maps show how the solar frequency distribution was in the state of Espírito Santo as well as in the municipality of Serra, where the research was developed. The main objective of the maps is to demonstrate how there is a variety of solar frequency in the different months of the year, even in a State that, being in Brazil, a country close to the equator, implies that an optimal frequency of solar incidence should occur during the entire year.

According to Planck,^23^ energy is directly proportional to frequency and the wavelength is inversely proportional to frequency. Thus, solar energy is closely related to the wavelength and, therefore, in regions that have greater solar energy availability, the wavelength will be predominantly shorter (with greater availability of UVB radiation). The evolution of solar energy availability over the months of the year, stratified by seasonality. The darker the area on the graph, the greater the incidence of sunlight, which means greater availability of sunlight under the demarcated region, favoring the conversion of vitamin D in the skin.

## DISCUSSION

This is the first study that was carried out in Brazil demonstrating the frequency of solar incidence in the country, correlating with vitamin D concentrations in children and adolescents, as well as demonstrating how important the contact of this population with the sun is, in order to activate vitamin D.

From the data visualized in this study, we emphasize the importance of tests related to vitamin D being compared between populations and even in a clinical way, being collected in the same periods of the year, taking into account the seasonal changes in solar incidence, and consequently in the metabolism of vitamin D in the human body.

Brazilian studies^1–4^ show an average of 50% of the population studied with vitamin D insufficiency, which corroborates the present study, which found 46.7%. Araújo et al.^4^ and Lopes et al.^3^ identified an association between overweight and reduced vitamin D concentration, the first study showed that, in males, adolescents classified as overweight/obese were 2.4 times more likely to have hypovitaminosis than eutrophic adolescents, this association was not found in the present study, since more than 70% of the population was classified as eutrophic and because it is an active population. According to Peters et al.,^2^ only 27.9% of the assessed adolescents practiced outdoor physical activity, with vitamin D insufficiency being found in 60% of the sample. In the present study, 74.3% of those eligible had been exposed to the sun through the practice of outdoor physical activity, and the prevalence of vitamin D insufficiency was 42.9% and we can observe that individuals who performed indoor activities had a prevalence of 8% more of hypovitaminosis D.

A major source of vitamin D for most humans comes from skin exposure to sunlight, typically between 10:00 and 15:00 h in the spring, summer, and fall.^6,15^ Vitamin D produced in the skin may last at least twice as long in the blood compared with ingested vitamin D.^24^ When an adult wearing a bathing suit is exposed to one minimal erythemal dose of UV radiation (a slight pinkness to the skin 24 h after exposure), the amount of vitamin D produced is equivalent to ingesting between 10,000 and 25,000 IU.^25^ A variety of factors reduce the skin’s production of vitamin D3, including increased skin pigmentation, aging, and the topical application of sunscreen.^25,26^ Typically, a human body requires about 3000–5000 IU vitamin D daily under the usual load.^27^

A study carried out in Poland with children aged 4–6 years analyzed the concentration of vitamin D in children and found that the best time of year to acquire vitamin D would be from May to September, with exposure having to take place between 10:00 and 15:00 h, for 15 min with the least amount of clothing, leaving the skin more exposed.^28^ The present study observed that children who spend more time doing outdoor activities are not less likely to have low concentrations of vitamin D (p<0.05), and the proportion of participants with inadequate vitamin D was 15% and 18% greater in the participants whose vitamin D was measured during spring and winter, respectively.

Most of the participants in this study had their data collected in the winter, and an explanation for this fact is that the students’ vacation period takes place in the summer, and that “Estação Conhecimento” observes the school vacation period; therefore, it closes its activities in mid-December and only returns in mid-February, and summer is determined as December to March. However, these data are also important since, theoretically, in Brazil, there would not be such a huge change in solar incidence, to the point of needing to supplement vitamin D as in Northern countries.^29^

The mountain region of Espírito Santo has a higher altitude, and this influences the increase in solar irradiance. However, the region is composed of much topographic unevenness, such as hills, which allows for the formation of clouds due to the humidity coming from the ocean, favoring the reduction of solar radiation. The coastal region of the State where the municipality of Serra is located has an annual average of solar irradiance between 4,701 and 4,850 Wh/m^2^/day.^30^ Solar irradiance in the region is more influenced by the solar zenith angle, which varies during the day and over the months. The months with the highest solar radiation value in the state of Espírito Santo were the months that represent the summer period in the southern hemisphere of the planet. In contrast, the months with the lowest incidence were the winter months.

The months that comprise the summer present greater solar irradiance and with that the production of vitamin D is accentuated. This is evident when the average concentration of vitamin D is compared with the values of solar irradiance. In the summer, the average concentration of vitamin D was 35.10 ng/mL while the solar radiation in that same period in 2017 reached 6,500–7,000 Wh/m^2^/day. Winter is the season of the year when the region receives less solar radiation and consequently the production of vitamin D is attenuated. The solar radiation presented maximum values between 5,001 and 5,500 Wh/m^2^/day. Such values show that an amount of energy was received from the sun, and this directly affects the average production of vitamin D in the same period, which was 28.24 ng/mL.

As for the limitations of the study, we can list the sample, as the collection of vitamin D was performed only once for each child, not being a longitudinal analysis but a random sample. However, the study proves to be important due to the survey of data carried out and associations made, other authors being able to carry out longitudinal studies and with representative samples of the population, using the same methodology addressed. Another limitation would be related to the effective time that the child or adolescent spends in the sun; however, the identification of the importance of this contact, through the present study, raises the question about the need to provide means for the child-juvenile population, to expose themselves to the sun more often, and to facilitate the activation of vitamin D in the body. In addition, we did not assess dietary intake of vitamin D, but it is known that vitamin D intake from food is incipient and the major factor that impacts its concentration is contact with the sun.

Even in a Latin American country, vitamin D concentration should be taken into account and frequently evaluated in children and adolescents. An important fact raised was the fact of collecting data from scientific studies or clinical analyses, according to seasonality, as it interferes with the concentration of vitamin D. It is extremely important that greater exposure to the sun is encouraged and that schools make available free time for children to be exposed to the sun, through outdoor physical activity, for example, and that governments provide means for this, since children and adolescents who are more exposed to the sun have a higher concentration of vitamin D.

This study initiates the discussion on the need to develop public policies for vitamin D supplementation in addition to public policies that encourage contact with the sun through outdoor activities for the specific public, even in a country that receives sun all year round.
